# Five crucial prognostic-related autophagy genes stratified female breast cancer patients aged 40–60 years

**DOI:** 10.1186/s12859-021-04503-y

**Published:** 2021-12-07

**Authors:** Xiaolong Li, Hengchao Zhang, Jingjing Liu, Ping Li, Yi Sun

**Affiliations:** Surgical Department of Breast Thyroid Surgery, Xuchang Central Hospital, No. 30 Huatuo Road, Weidu District, Xu Chang, 461600 Henan Province China

**Keywords:** Autophagy genes, SERPINA1, HSPA8, HSPB8, MAP1LC3A, DIRAS3

## Abstract

**Background:**

Autophagy is closely related to the progression of breast cancer. The aim at this study is to establish a prognostic-related model comprised of hub autophagy genes (AGs) to assess patient prognosis. Simultaneously, the model can guide clinicians to make up individualized strategies and stratify patients aged 40–60 years based on risk level.

**Methods:**

The hub AGs were identified with univariate COX regression and LASSO regression. The functions and alterations of these selected AGs were analyzed as well. Moreover, the multivariate COX regression and correlation analysis between hub AGs and clinicopathological parameters were done.

**Results:**

Totally, 33 prognostic-related AGs were obtained from the univariate COX regression (*P* < 0.05). SERPINA1, HSPA8, HSPB8, MAP1LC3A, and DIRAS3 were identified to constitute the prognostic model by the LASSO regression. The survival curve of patients in the high-risk and low-risk groups was statistically significant (*P* < 0.05). The 3-year and 5-year ROC displayed that their AUC value reached 0.762 and 0.825, respectively. Stage and risk scores were independent risk factors relevant to prognosis. RB1CC1, RPS6KB1, and BIRC6 were identified as the most predominant mutant genes. It was found that AGs were mainly involved in regulating the endopeptidases synthesis and played important roles in the ErbB signal pathway. SERPIN1, risk score was closely related to the stage (*P* < 0.05); HSPA8, risk score were closely related to T stag (*P* < 0.05); HSPB8 was closely related to N stag (*P* < 0.05).

**Conclusions:**

Our prognostic model had the relatively robust predictive ability on prognosis for patients aged 40–60 years. If the stage was added into the prognostic model, the predictive ability would be more powerful.

**Supplementary Information:**

The online version contains supplementary material available at 10.1186/s12859-021-04503-y.

## Introduction

Autophagy is a natural phenomenon that regulates cell metabolism inside. Under normal physiological conditions, the immune cell autophagy process could eliminate senile organelles and abnormal long-lived proteins in the body, which will be conducive to maintaining immune cell homeostasis. However, a stress stimulus, could result in cell autophagy working in preventing the accumulation of toxic or carcinogenic damaged proteins and organelles and inhibiting simultaneously cell cancelation. If it occurred dysfunctional autophagy process, a tumor could be coming soon [1]. Therefore, during the oncogenesis process, autophagy has a double function with promotion and suppression.It has been implicated that tumorigenesis is closely related to cell autophagy [2].

Breast Cancer(BC) is one of the most common malignancies for women. The 5-year survival rate of patients is low and it is easy to turn out especially on middle-aged 40–60 years women. Given it is prone to distant metastases, early diagnosis is of the great significance to the prognosis of BC patients [3]. So far, conventional clinical evaluation means, such as TNM stage and pathological classification, are tough to precisely speculate patients’ prognosis in fact and offer clinical doctors more accurate treatment choices. In our study, analyzing the RNA-sequence data onto the autophagy-genes (AGs) from female BC patients at a high incidence age, a prognostic model composed of several signatures was conceived, more accurately dividing BC patients into high-risk and low-risk groups, which will benefit to clinicians to target Patients in various risk levels and to adopt corresponding individualized strategies.

## Methods

### Download data

The Illumina HTSeq-FPKM data (mRNA gene expression data) and clinical data of female BC patients, whose ages were 40–60 years and whose pathological types were ductal and lobular tumors, were downloaded from The Cancer Genome Atlas (TCGA, https://www.cancer.gov/about-nci/organization/ccg/research/structural-genomics/tcga,) and Gene Expression Omnibus (GEO, https://www.ncbi.nlm.nih.gov/gds/) databases. For mRNA expression data, ID must be converted to a gene name, and then these genes expression data were sorted into a gene matrix pattern. Clinical data included age,gender,grade,stage,T,N,M,survival status and survival time. Genes with an expression value of 0 and cases with incomplete clinical data are deleted from our model. We can find AGs recommended in Human Autophagy Database: HADb (http://autophagy.lu/).

### Filter out prognostic AGs and adventurous clinical factors for constructing the model

Cox regression analysis takes survival outcome and survival time as dependent variables and can analyze how many factors have an influence on survival at the same time. So it is widely used in clinical prognosis analysis. The characteristic of LASSO regression is to perform a variable selection and a complexity regularization while it is used to fit a generalized linear model. LASSO regression can process high-latitude data and select the most valuable key factors, to avoid the model overfitting caused by lots of variables included in the model. The univariate and multivariate COX regression (Cox proportional-hazards model) was performed to screen prognostic AGs by R software package “survival” [4]. While the Least Absolute Shrinkage and Selection Operator (LASSO regression) was to construct the model of autophagic gene prognosis and to calculate patients risk score through R software package “glmnet”.Through including clinical and pathological factors, the multivariate COX regression could filter out independent risk factors, which were jointly incorporated into the model construction.

### The various indexes evaluated the practicality of this predictive model

The performance of the AGs model was assessed through plotting survival curves by R package “survival”. With the median of risk score as the boundary, patients above the median were classified into the high-risk group, and patients below the median were classified into the low-risk group. A Kaplan–Meier survival curve was drawn to observe the survival rates in both groups, while the Log Rank method was to compare whether there were significant differences in survival rates of the two groups.

The Receiver Operating Characteristic Curve (ROC) was used to evaluate the predictive ability of the AGs model for a 3-year or 5-year survival rate by R package “survivalROC”. We analyzed the main functions of prognostic-related AGs through gene ontology (GO) and Kyoto Encyclopedia of Genes and Genomes (KEGG) by R package “enrichplot”, “ggplot2”. Gene alterations were detected in cBioportal database(http://www.cbioportal.org/). Simultaneously, a correlation analysis was done between hub AGs and clinical factors.

### Externally independent validation

The generalization ability of our risk score model was verified in GEO [5]. On the protein level, we validated the expressions of five hub genes in normal tissues and tumor tissues through Human Protein Atlas (HPA, https://www.proteinatlas.org). The database provided immunohistochemistry (IHC) results using a tissue microarray (TMA)-based analysis of the corresponding proteins in PC patients and normal tissues. IHC staining for each gene was done using the same antibodies in tumor tissues as in normal tissues. By comparing the staining pictures of two kinds of tissues, we can generally judge the expression of key genes on protein level among them.

## Statistics

R software 3.4.2 was used for statistic analysis and drawing pictures. It can perform Kaplan–Meier, LASSO and multivariate COX regression, ROC, calibration curves, as well as correlation analysis. MedCalc software 19.3.1 was used for carrying out DeLong's test to make the comparison of multiple ROC. *P* < 0.05 was regarded as existed significant difference statistically.

## Results

### Screening prognostic-related AGs by univariate COX regression

**Fig. 1:**
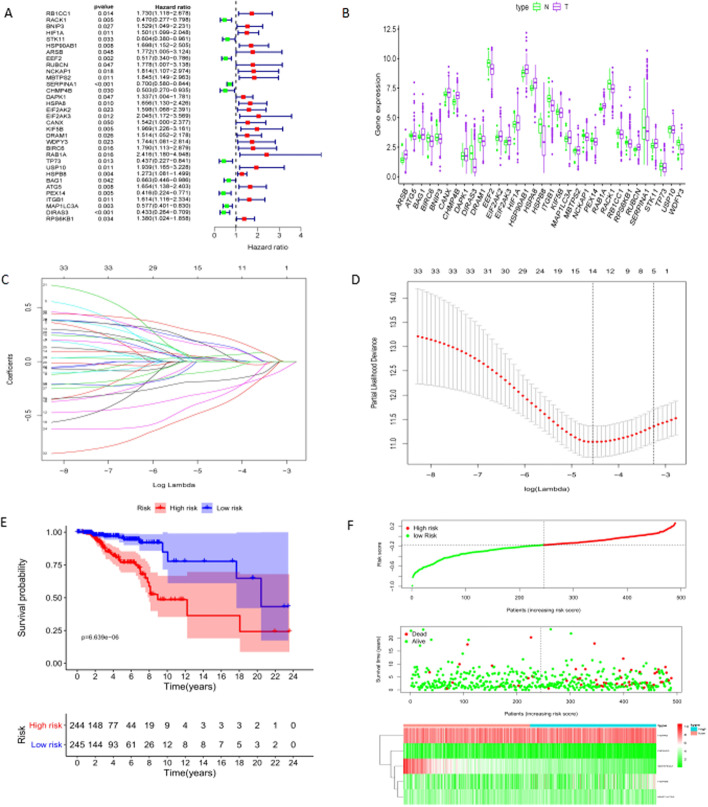
Establish the prognostic-related risks core model comprised of five mRNA genes. (**A**) Univariate Cox regression analysis was to screen potential autophagy genes related to prognosis.red scale: upregulated genes; green scale: downregulated genes. (**B**) Validate selected autophagy genes expression in the normal group(N) and tumor group(T). (**C**) LASSO regression filtering out the most representative five hub autophagy genes. The down horizontal axis represented the model coefficient ratio(lambda), and the upper one represented variables numbers. The vertical axis represented the coefficient. Each colored solid line represented a gene variable coefficient. A vertical dashed line was drawn based on the horizontal axis at lambda.1se, as D. At lambda.1se, the vertical dashed line only intersected with five colored solid lines. It was indicated that five crucial genes were selected to be the most significant impact factors on prognosis. (**D**) tenfold cross-validation was to identify the minimal lambda and its 1-standard error away(1-se) with the least core autophagy genes. The data set were divided into ten parts, alternately 9 parts as training data and 1 part as test data. Left vertical dashed line: at minimal lambda 0.01053932; Right vertical dashed line: at 1-se minimal lambda 0.03532362. (**E**) The survival rate comparison between high risk score and low-risk risk score patients. (**F**) The risk plot for high risk score and low risk score patients. Vertical dashed line is the boundary of both group patients

To download the RNA-sequence data and survival data onto 501 female BC patients whose ages were 40–60 years old from the TCGA database, the expression data of the whole 194 AGs were extracted. The univariate Cox regression analysis was performed on 501 BC samples, and 33 prognostic-related AGs were obtained, in which 23 up-regulated and ones and 10 down-regulated ones (Fig. 1A). Simultaneously, there were significant differences in these genes expressions of healthy women and female BC patients (*P* < 0.05) (Fig. 1B).

### The five hub AGs identified by LASSO regression

LASSO regression is a compressed estimation method. It obtains a more refined model by constructing a penalty function (Lambda), which makes it compress some regression coefficients. That is to force the sum of absolute values of coefficients to be less than a certain fixed value and simultaneously to set some regression coefficients to zero. Therefore, the advantage of subset shrinkage is retained, and it is a biased estimation for processing data with multicollinearity. Lambda was decided through cross-validation: the Lambda value with the smallest error is selected to refit the model with all the data. Lambda.min refers to the one where we can obtain the mean value of the least target variables among all the λ values. And lambda.1se refers to the lambda value of the simplest model within a variance range of lambda.min. As the λ value reaches a certain value, continuous increasing the number of independent variables can not significantly improve the model performance. Lambda.1se gives a model with excellent performance but the least number of independent variables. The five AGs were selected from 33 prognostic-related AGs by LASSO regression, whose risk scores were calculated based on their respective correlation coefficients for risk stratification of BC prognosis (Fig. 1C). At lambda.1se, there were five AGs screened, Serpin family A member 1 (SERPINA1), Heat shock protein family A (Hsp70) member 8 (HSPA8), Heat shock protein family B (small) member 8 (HSPB8), Microtubule-associated protein 1 light chain 3 alpha (MAP1LC3A), and DIRAS family GTPase 3 (DIRAS3) (Fig. 1D). Formula: Risk score = SERPINA1 expression levels* (-0.107130438454055) + HSPA8 expression levels* 0.0342643673417569 + HSPB8 expression levels* 0.0320959903903294 + MAP1LC3A expression levels * (-0.0206809753743301) + DIRAS3 expression levels * (-0.0415014293356393). On the protein level, we validated these hub genes expressions of normal tissues and tumor tissues through the HPA database (Additional file 1).HSPA8 and HSPB8 expressed higher in tumor tissues than that in normal tissues, while SERPINA1 and DIRAS3 expressed higher in normal tissues against tumor tissues. MAP1LC3A was weakly expressed in tumor and normal tissues (Additional file 1: Fig. S1).

### The differentiated survival curve depended on five-AGs-risk score between the high-risk group and the low-risk group

The risk score was constructed based on five crucial AGs related to prognosis. R software package “survival” was used to calculate a prognostic-AGs risk score of every patient in our model. To more accurately assess the predictive value of the risk score, we divided all patients into a high-risk group (> -0.2) and a low-risk group (< = -0.2)at a risk score median (-0.2). The difference in survival curves between the high-risk group and the low-risk group was statistically significant (*P* = 6.639e-06). The survival rate of patients with high-risk scores was significantly lower than that of patients with low-risk scores (Fig. 1E). SERPINA1, MAP1LC3A, DIRAS3 were down-regulated in a high risk group and up-regulated in a low risk group, which were potentially tumor suppressor genes; HSPA8, HSPB8 were up-regulated in a high risk group and down-regulated in a low risk group, which were probably promoting oncogenes. The number of dead patients in the high risk group was significantly more than that in the low risk group (Fig. 1F).

### Identification of the independent risk prognostic factors

**Fig. 2:**
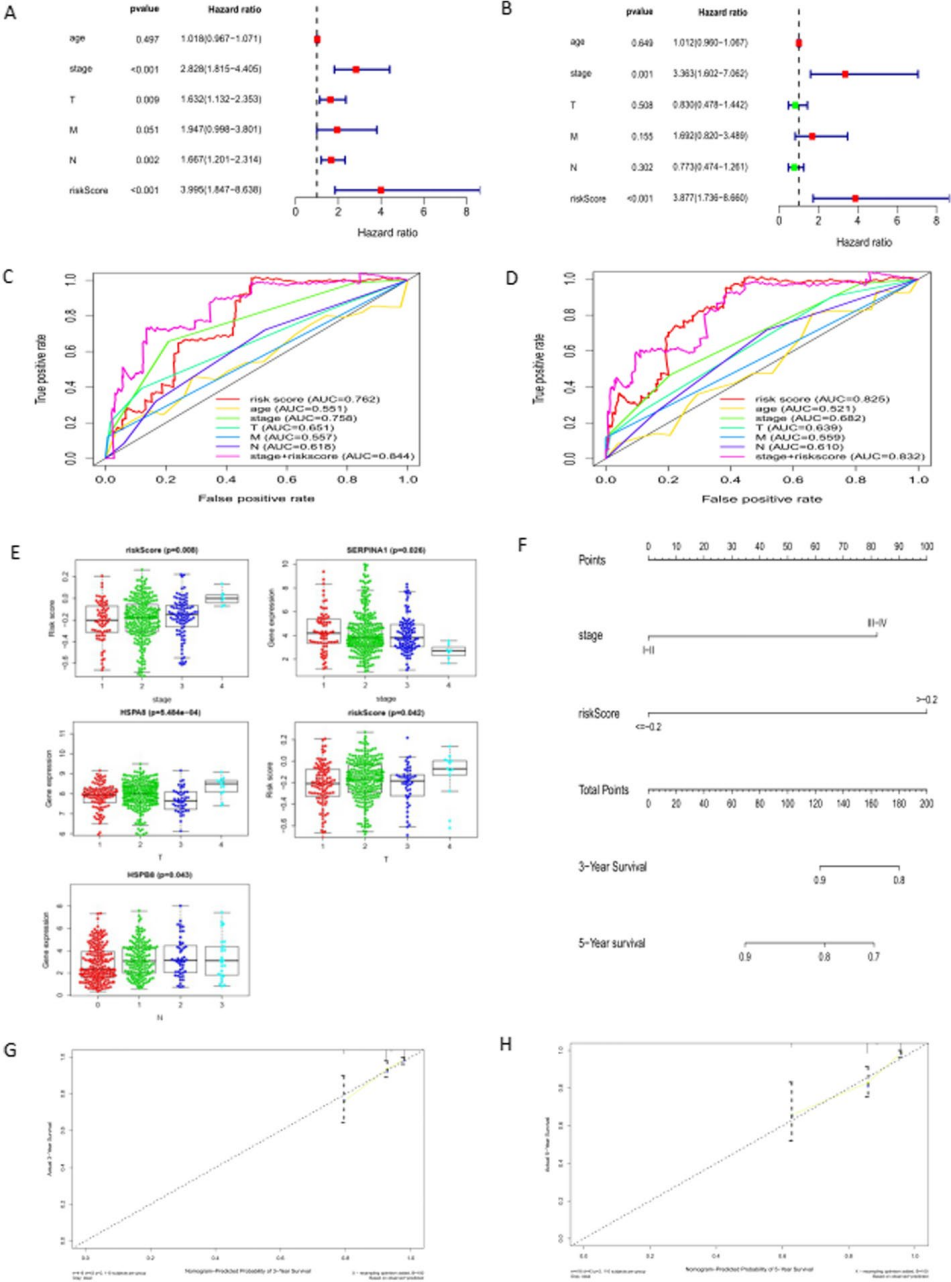
Clinical and pathological factors involved in constructing the risk score model. (**A**) Univariate COX regression. (**B**) Multivariate COX regression.red scale:Hazard ratio > 1;green scale:Hazard ratio < 1. (**C**) ROC for 3-years survival rate. (**D**) ROC for 5-years survival rate. (**E**) The correlation analysis of critical autophagy genes with clinical factors. All patients are divided into two groups according to clinical characteristics. The R package “beeswarm” was used to do a t-test for comparing five hubs genes expression data and risks core with clinical characteristics among two groups. *P* < 0.05 was plotted and suggested that there were significant differences between them. In this figure, the middle horizontal line represented mean; the upper and down ones represented standard deviation. (**F**) Nomogram built by all high-risk factors. The R package “survival” was used to draw a point line through performing cox regression. Each impact factor corresponds to the corresponding score on the point line. All the points were added to get a total point. At the total point, a vertical line intersected with the survival rate line. Then, we could obtain the survival rate of each patient. (**G**) Calibration curve for 3-years prediction. (**H**) Calibration curve for 5-years prediction

The univariate and multivariate COX regression analysis on various clinical-pathological parameters was done, such as age, stage, T, M, N, and risk score, to screen independent risk factors affecting prognosis. Univariate Cox regression analysis suggested that stage, T, N, and risk score were risk factors affecting the prognosis of BC patients (*P* < 0.05, HR > 1) (Fig. 2A), while multivariate Cox regression suggested that stage and risk score were independent risk factors(*P* < 0.05, HR > 1) (Fig. 2B). The AUC(Area Under the Curve) values of the risk score reached 0.762 in three years and 0.825 in five years. The AUC of the stage alone in three years also reached 0.758. However, for five years, the AUC value was 0.682. The AUC value of stage combined with risk score was 0.844 in three years and 0.832 in five years (Fig. 2C, D). It was implicated that stage and risk score was more accurate than alone risk score on predictive ability. DeLong's test suggested the predictive ability of stage + risk score has more robust than that of an alone stage or alone risk score (*P* < 0.05) in Fig. 2C or D.

### Correlation analysis between the five hub AGs expression and clinicopathological parameters

We attempted to discover whether the five key AGs and their risk score were correlated with these clinicopathological factors. SERPIN1, risk score and stage were closely related (t = 9.268, *P* = 0.026; t = 11.79, *P* = 0.008); HSPA8, risk score and T stag is closely related (t = 17.535, *P* = 0.484e-04;t = 8.185, *P* = 0.042); HSPB8 is closely related to N stag (t = 8.136, *P* = 0.043) (Table 1, Fig. 2E).

**Table 1:** The correlation coefficients between key autophagy genes and clinicopathological factors (r2, P)

Gene	Age	Stage	T	M	N
*SERPINA1*	16.975 (0.655)	9.268 (0.026)*	4.484 (0.214)	0.58 (0.571)	1.761 (0.623)
*HSPA8*	25.36 (0.188)	0.404 (0.939)	17.535 (5.484e−04)*	1.373 (0.192)	0.386 (0.943)
*HSPB8*	15.945 (0.720)	2.925 (0.403)	3.336 (0.343)	− 0.322 (0.753)	8.136 (0.043)*
*MAP1LC3A*	28.06 (0.108)	0.354 (0.950)	3.681 (0.298)	− 1.758 (0.101)	1.105 (0.776)
*DIRAS3*	29.925 (0.071)	2.948 (0.400)	7.174 (0.067)	0.514 (0.616)	1.853 (0.603)
*riskScore*	14.958 (0.779)	11.79 (0.008)*	8.185 (0.042)*	− 0.313 (0.759)	3.355 (0.340)

* *P* < 0.05, those key autophagy genes were highly correlated with clinicopathological factors

### The performance of clinical prognostic model based on five hubs AGs

Double independent prognostic risk factors, stage, and risk score, were included in an established nomogram (Fig. 2F). C-index reached 0.745 (95% CI, 0.709- 0.778). At the same time, we plotted the calibration curve and further evaluated the accuracy of our model. Its results were showed that there was no statistically significant difference between the model-predicted value and the true one (Fig. 2G, H). The multi-factor ROC illuminated that the risk score has the most robust predictive ability for BC prognosis among all clinicopathological factors.

### The function analysis of prognostic-related AGs

**Fig. 3:**
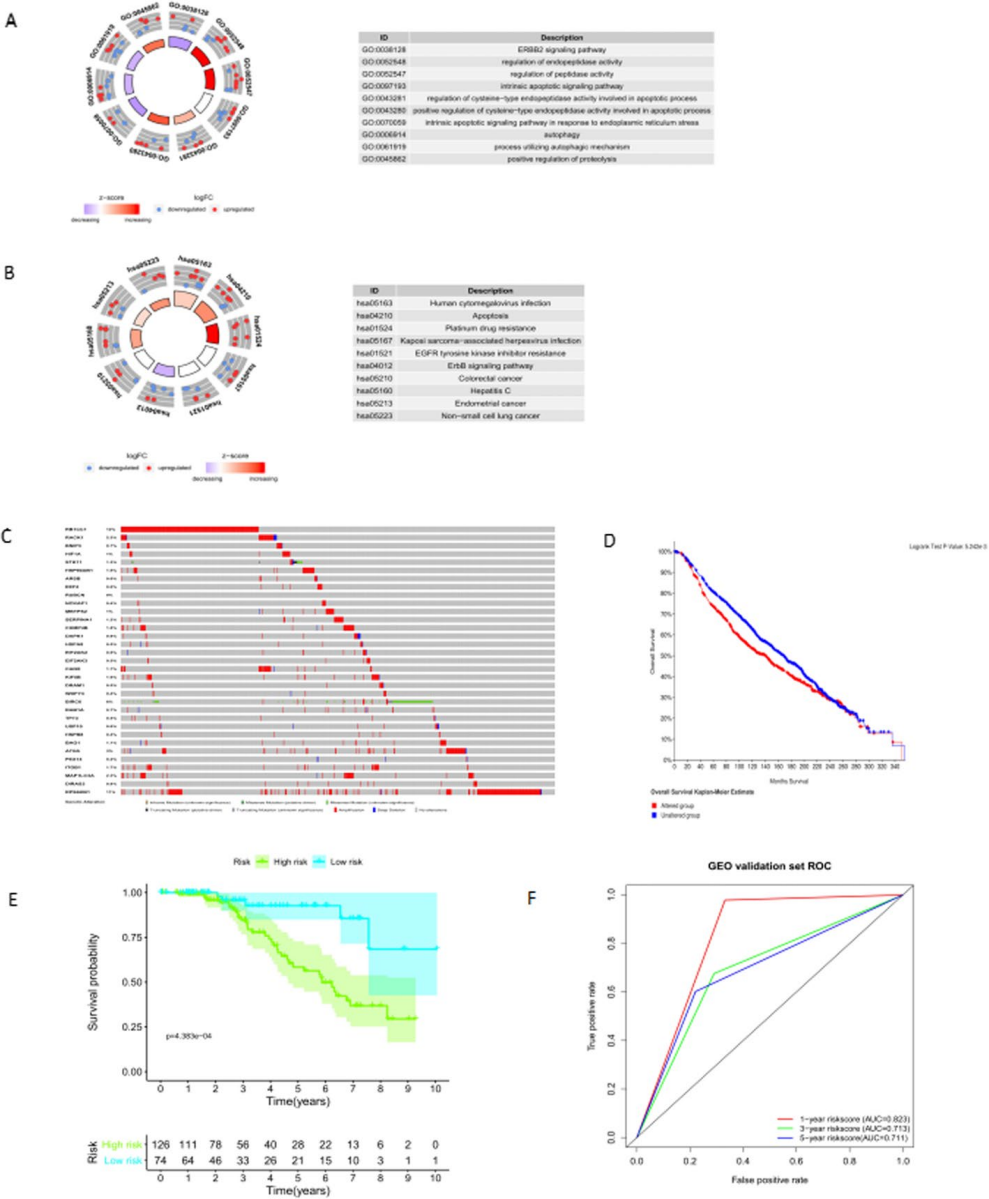
The genes function enrichment analysis and external validation. (**A**) GO circle of genes function. (**B**) KEGG circle of pathway enrichment analysis. (**C**) Oncoprint reflecting autophagy genes alteration. (**D**) Survival differentiation among an altered group and unaltered group. (**E**) Survival Service differentiation between high and low risk score patients in GEO set. (**F**) ROC assessment in GEO independent validation cohort

The prognosis-related AGs of BC selected by univariate COX regression were performed GO and KEGG function enrichment analysis. It was found that up-regulated AGs were mainly involved in the regulation of endopeptidases in vivo (Fig. 3A), while down-regulated AGs were mainly involved in adjusting ErbB signaling (Fig. 3B). We have also taken the cBioPortal database (https://www.cbioportal.org/) of BC patients (METABRIC, Nature 2012 & Nat Commun 2016) with mutations and putative copy-number changing from DNA copy to verify the genetic alteration. As a consequence, 815 (38%)s of 2173 patients/samples had occurred genetic changes, in which RB1CC1 (12%), RPS6KB1 (11%), and BIRC6 (6%) had the most genetic variety (Fig. 3C). There was a significant discrepancy in a survival rate for the genetically altered group and the unaltered group (*P* = 5.242e-3), and the prognosis of patients in the genetically modified group was worse (Fig. 3D).

### The external validation of the AGs prognostic models on the GEO database

To confirm the practicality of the five-hub-AGs prognostic model, we downloaded GSE96058, with platform GPL11154, Series Matrix File (s), and RNA sequence data from GEO. Randomly select 200 patients aged 40–60 years from 3273 samples, and calculate five-AGs-risk score. A cut-off value risk score of -0.2, patients were divided into a high-risk group and a low-risk group. The evident difference existed on both groups, and the survival rate of high-risk patients was significantly lower than that of low-risk groups (*P* = 4.383e-04) (Fig. 3E). The ROC curve of the verification group showed that the risk score has relatively good generalization ability (Fig. 3F).

## Discussion

Autophagy is a complex immune process regulated genetically, in which autophagy-related genes are involved. It can supervise genome alteration and impair genomic instability [6]. Carcinogenesis is subjective to genetic mutations. The role of AGs in tumorigenesis and progression is extremely complicated and its function is likely to be multiple in diverse stages of cancer progression [7]. In the early stage, autophagy might preserve cell survival through cellular catabolism. The expression of AGs might hinder cells proliferation with cancer-associated mutation tendency, preventing tumorigenesis by promoting senescence. However, once the tumor has been exhibited, cancer cells can protect themselves by autophagy. During the late stage of tumorigenesis, autophagy can increase oxidative stress, thereby facilitating genomic instability and malignant transformation.

The 5-year survival rate of BC patients is about 26%, nearly 12% of whom eventually develops to metastatic disease, meaning cancer cells disseminate to other organs from breast [8]. with a median survival time of 18–30 months, it would induce poor prognosis and high mortality [9, 10]. To date, some scholars have contributed to the relationship between BC and autophagy. Vega-Rubín-de-CelisS et al. [11] have explored the regulated mechanism of HER2 to autophagy in vita, and the impacts of genetic and pharmacological strategies on HER2-driven BC proliferation enhancing autophagy in vivo. It was found that HER2 mediated inhibition for Beclin 1 autophagy might be beneficial to HER2-mediated tumorigenesis, and the strategies blocking HER2 / Beclin 1 binding or increased autophagy were promising on curing HER2-positive BC patients. Timothy Marsh et al. [12] temporarily removed essential autophagy regulators during cancer progression by BC model. Although AGs ablation intensively weakened the growth of primary breast tumors, impaired autophagy would promote spontaneous metastasis and causes metastatic tumor cells appear to be large ones. Their research results suggested that NBR1 autophagy-dependent administration was a crucial determinant of the metastatic process. Yeo et al. [13] have verified whether macrophage autophagy inhibition was effective in BRCA1-deficient breast tumors. By the K14-Cre transgene, they created mice with conditionally deleted basic AGs, Rb1cc1, Brca1, and Trp53. Finally, they found that autophagy suppression could increase profits of dimethyldibase treating BRCA1-deficient BC. Walker et al. [14] discovered the protective mechanism of BC cells under the condition of glucose deprivation. Nrf2 signaling played a vital role in protecting BC cells during glucose deprivation-induced autophagy through its antioxidant activity. Ji et al. [15] examined the effects of NVP-BEZ235 on viability, apoptosis, and autophagy in BC cell lines. They also tested how NVP-BEZ235 made an influence on the expression of p-AKT, p-mTOR, and p-70S6K in the pathway PI3K / AKT / mTOR. The results showed us that NVP-BEZ235 significantly suppressed BC cells proliferation and induced apoptosis and autophagy in MCF-7 cells. Lozy et al. [16] found that low-expressed BECN1 was associated with ERBB2 overexpression in BC, which suggested that BECN1 deletion and ERBB2 overexpression might functionally interact during breast tumorigenesis. ERBB2 overexpression repressed autophagy responds to stress on mouse mammary glands and Human BC cells. ERBB2-driven breast tumorigenesis was closely correlated with functional autophagy inhibition. Hamurcu et al. [17] detected the role of FOXM1 in regulating autophagy in TNBC (triple-negative BC) cells. FOXM1 expression was up-regulated during the process of autophagy induction. They found that repressing FOXM1 could inhibit autophagy induced by starvation and rapamycin and the expression of major autophagy regulators, LC3 and Beclin-1.

Notably, despite many studies have explored the role of autophagy in tumorigenesis, most work has focused on analyzing a single autophagy gene in one or two cell lines or animal models. Few people have been concerned about the meaning of prognostic datasets with high-throughput sequence expression Profiles in BC. With the coming of high-throughput "omics" data, it is now likely to study expression patterns of global AGs and their involvement in predicting BC outcomes.

In our study, prognostic-related AGs were analyzed by high-throughput expression profile. Eventually, SERPINA1, HSPA8, HSPB8, MAP1LC3A, and DIRAS3 were identified to be hub AGs related to prognosis and to calculate the patients’ risk score. When a patients’ risk score was more than -0.2, the patient would be regarded to be at high risk. When his or her risk score was less than -0.2, we consider the patient to be at low risk. Besides it, we have compared the survival rates of patients at high risk and patients at low risk, which existed significant differences. The AUC value of the ROC for evaluating the 5-year survival rate of BC patients was 0.825. If another independent risk factor, stage, was added into our model, the AUC value would reach 0.832.

Futhermore, the relationship between these five AGs and BC had been studied by other scholars as well. Chan et al. [18] discovered that ER was constitutively activated, resulting in binding with the SERPINA1 gene and up-regulation of SERPINA1 expression. High expression of SERPINA1 might herald better clinical outcomes of ER+ and ER+/HER2+ patients. Yu et al. [19] believe that HSPA8 (heat shock protein 70) was a target for anti-cancer therapy. Overexpression of HSPA8 was usually observed in BC. Inhibition of HSPA8 expression could induce apoptosis of BC cells. Piccolella et al. [20] found that HSPB8 was highly expressed in triple-positive hormone-sensitive BC cells (MCF-7) and was involved in regulating cells cycle and cell migration. Othman et al. [21] uncovered that, compared with normal tissues, a large number of BC tissues expressed the MAP1LC3A protein with strong immunoreactivity. It was indicated that MAP1LC3A was likely to play an indispensable role in the progression of BC. Nowak et al. [22] revealed that DIRAS3 could regulate cells cycle and impair the growth and movement of cancer cells, all of which might be indirectly dependent on the interaction with STAT3. The down-regulation of DIRAS3 expression was related to the progression of BC, while the restoration of DIRAS3 expression could restain cell proliferation and invasiveness. We also performed the functional analysis of prognostic-related AGs and found that they were mainly engaged in the regulation of endopeptidase and ERBB2 signaling pathways. Sisinni et al. [23] focused on endoplasmic reticulum stress and the relationship between apoptosis and autophagy in human BC. They thought that endoplasmic reticulum stress resulted in levels and activities alteration of key regulators about cell survival and autophagy, promoting to increase endopeptidase synthesis. Moreover, the ERBB2 signaling pathway was a critical pathway to regulating BC autophagy [16].

We also compared other BC prediction models to ours. It was implicated that our model was simpler and easier to operate than theirs, and the AUC value of reflecting predictive ability was relatively higher. Shen et al. [24] investigated that 11-lncRNA signature was a novel and significant prognostic element independent among multiple clinical and pathological parameters. The TIMER database showed that the 11-lncRNA prognostic characteristic was relevant to the infiltration of immune cell subtypes in BC. Wu et al. [25] identified vital pathways revealing potential molecular mechanisms and core genes probably used as BC biomarkers through WGCNA( Weighted Correlation Network Analysis). The three central genes, CBR3, SF3B6, and RHPN1, might play important roles in malignancy cancer transformation. Gu et al. [26] mentioned that eight AGs, BCL2, BIRC5, EIF4EBP1, ERO1L, FOS, GAPDH, ITPR1, and VEGFA, were ought to be designated as prognostic markers for autophagy-associated BC. Lin et al. [27] initially found that 27 ARGs (autophagy-related genes) were associated with an overall survival rate of BC. The prognostic-related ARGs signature established by the Cox regression consisted of 12 ARGs with a 5-year survival rate AUC 0.742.

Of course, our autophagy-related risk score model has some limitations. The expression of these five hub genes is supposed to be verified in other independent datasets, and the practicality of this predictive model should be detected through feedbacks from clinical applications. LASSO is a well-known non-robust variable selection method that cannot handle skewed distributions especially under heterogeneous prognostic outcomes. Probably, we could try the R package “regnet” for a direct comparison. In addition, researches on the function of AGs are particularly meaningful. For instance, how autophagy deletion or autophagy inhibition promotes the gene expansion and growth of BC cells, and the interaction mechanism between its functions remains to be investigated, which has great significance for the treatment and prognosis of BC patients. The functional status of autophagy and its pharmacological manipulation of BC treatment will become novel targeted treatment options.

## Conclusions

we have constructed a prognostic model for predicting the survival rate of BC patients composed of five essential AGs, attempting to provide guidance of clinicians on making up personalized treatment strategies corresponded to different risk level patients. The autophagy signature represents a promising biomarker for assessing the overall survival rates of BC patients.

## Supplementary Information

**Additional file 1: Fig. S1**. The expression of the five hubs prognostic AGs on the protein level. The database provided immunohistochemistry (IHC) results using a tissue microarray (TMA)-based analysis of the corresponding proteins in PC patients and adjacent normal tissues. IHC staining for each gene was done using the same antibodies in tumor tissues as in normal tissues. However, the estimation of protein expression could not be performed.

## Data Availability

The AGs expression data are derived from TCGA gdc (https://portal.gdc.cancer.gov/). For the “cases” column, primary site (breast),program (TCGA),Gender(female) and age at diagnosis(40–60 years) were selected in turn. For the “files” column, data category(transcriptome profiling), data type(gene expression quantification),workflow type(HTSeq-fpkm) were selected in turn. Then, click “add all files to cart” and download “metadata” and “manifest” and “cart”. As for survival data, other selections were the same to gene expression data, besides data category( clinical) and data format(bcr xml).The GEO database (https://www.ncbi.nlm.nih.gov/geo/) provided a dataset (accession number: GSE96058) for a further validation. A platform GPL11154 was selected and a Series Matrix File(s) was downloaded.

## Abbreviations

AGs: Autophagy genes
BC: Breast Cancer
TCGA: The Cancer Genome Atlas
GEO: Gene Expression Omnibus
LASSO: The Least Absolute Shrinkage and Selection Operator
ROC: The Receiver Operating Characteristic Curve
GO: Gene ontology
KEGG: Kyoto Encyclopedia of Genes and Genomes
HPA: Human Protein Atlas
IHC: Immunohistochemistry
SERPINA1: Serpin family A member 1
HSPA8: Heat shock protein family A (Hsp70) member 8
HSPB8: Heat shock protein family B (small) member 8
MAP1LC3A: Microtubule-associated protein 1 light chain 3 alpha
DIRAS3: DIRAS family GTPase 3
