# Supplementary material for: Five crucial prognostic-related autophagy genes stratified female breast cancer patients aged 40–60 years
Source: BMC Bioinformatics. 2021 Dec 7;22:580. doi: 10.1186/s12859-021-04503-y (PMC8650421; doi:10.1186/s12859-021-04503-y)
Supplement: Supplementary file 1 — Additional file 1: Fig. S1. The expression of the five hubs prognostic AGs on the protein level. The database provided immunohistochemistry (IHC) results using a tissue microarray (TMA)-based analysis of the corresponding proteins in PC patients and adjacent normal tissues. IHC staining for each gene was done using the same antibodies in tumor tissues as in normal tissues. However, the estimation of protein expression could not be performed. [file 12859_2021_4503_MOESM1_ESM.pdf]

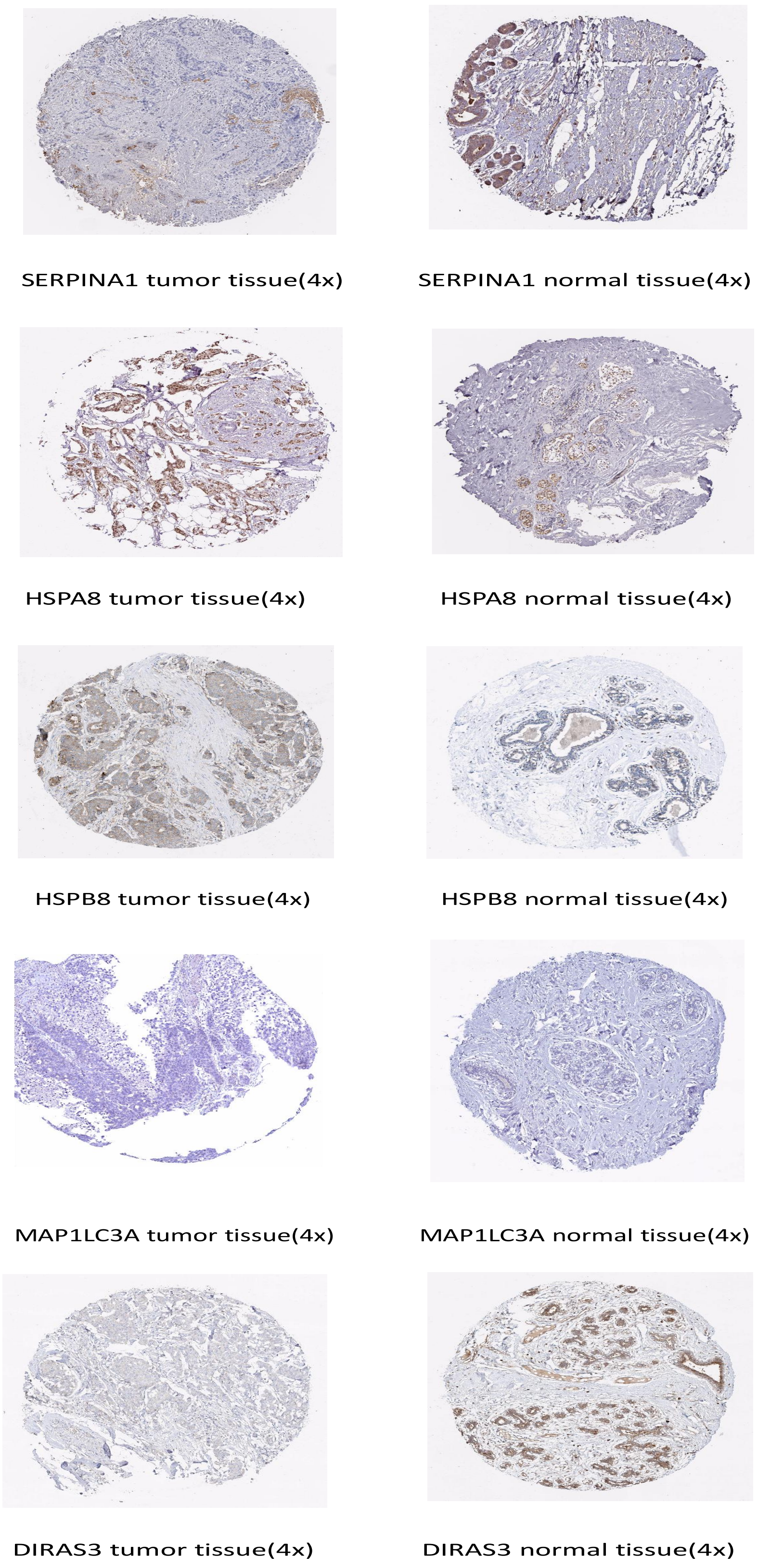

Figure S1 The expression of the five hub prognostic AGs on the protein level
